# AI-initiated second opinions: a framework for advanced caries treatment planning

**DOI:** 10.1186/s12903-024-04551-9

**Published:** 2024-07-10

**Authors:** Tudor Dascalu, Shaqayeq Ramezanzade, Azam Bakhshandeh, Lars Bjørndal, Bulat Ibragimov

**Affiliations:** 1 Department of Computer Science, University of Copenhagen, Copenhagen, Denmark; 2 Department of Odontology, University of Copenhagen, Copenhagen, Denmark

**Keywords:** Artificial intelligence, CAD, Caries, Computer vision/convolutional neural networks

## Abstract

Integrating artificial intelligence (AI) into medical and dental applications can be challenging due to clinicians’ distrust of computer predictions and the potential risks associated with erroneous outputs. We introduce the idea of using AI to trigger second opinions in cases where there is a disagreement between the clinician and the algorithm. By keeping the AI prediction hidden throughout the diagnostic process, we minimize the risks associated with distrust and erroneous predictions, relying solely on human predictions. The experiment involved 3 experienced dentists, 25 dental students, and 290 patients treated for advanced caries across 6 centers. We developed an AI model to predict pulp status following advanced caries treatment. Clinicians were asked to perform the same prediction without the assistance of the AI model. The second opinion framework was tested in a 1000-trial simulation. The average F1-score of the clinicians increased significantly from 0.586 to 0.645.

## Introduction

Dental diseases are among the most common disorders, disrupting one’s life with discomfort and pain [1, 2]. Although treatable with expert care, they pose major health problems, especially in developing countries, due to costs and work overload [2]. Over the past decade, AI-based systems have shown great potential for improving dental diagnostic accuracy and treatment planning [3, 4]. A particular use case of AI in dentistry concerns detection, classification, and care planning for carious lesions [4–8]. According to the American Dental Association (ADA), it can assist in the early detection of enamel caries, thereby enabling more minimally invasive treatment approaches [9]. Additionally, AI can quantify the percentage of enamel re/demineralization over time, enabling lesion progression forecasting [9]. Currently, the advanced caries treatment is in focus and less invasive excavation strategies are starting to be evaluated and adopted [10, 11], but there is a gap between the advocated excavation types case selection methods [12] and preferred performance in general dental practice [11]. Decades ago, the point of leaving caries behind using two stages to avoid pulp exposure was shown to be effective as opposed to performing complete excavation [13]. Further, employing this two-stage method has been shown to arrest the progression of the retained caries [14]. This evidence leads to the question of whether removing all infected dentin before sealing the tooth is necessary [15]. The most recent systematic Cochrane review confirmed that selective carious removal in one stage appears to be as effective as stepwise in two stages [10], making selective carious tissue removal the first treatment of choice [10, 16]. Notably, detailed analysis reveals that while evidence supports selective tissue removal for radiographically advanced lesions in the pulpal quarter, it remains limited, particularly in terms of long-term follow-up [10]. Considering this, the report from ADA [16] indicates that when selective tissue removal is not practical, both stepwise and non-selective carious tissue removal are acceptable treatment alternatives. The overarching aim of the present work is to investigate the potential of AI-initiated second opinions, demonstrated in enhancing advanced caries treatment planning and outcome prediction, an area that continues to present challenges.

Although numerous studies highlighted the potential of AI in solving dental tasks, few AI-based methods have been tested in clinical experiments with a focus on AI-dentist interaction [4, 17]. These types of studies are important not only because clinicians often exhibit a lack of trust in computer-based diagnoses [18, 19]. Mertens et al. [4] conducted a randomized controlled trial to assess the efficacy of AI-assisted detection of proximal caries. The number of intelligent systems approved by the US Food and Drug Administration (FDA) in dentistry, although growing [20], is an order of magnitude lower than those approved for use in cardiovascular, pulmonology, and neurology-related applications. The FDA states that AI solutions must demonstrate a validated correlation between their and their targeted conditions [9]. These solutions should employ data that is not only validated but also follows privacy and security protocols [9]. Further research is needed to assess the impact of AI-practitioner and AI-patient interactions in real-world conditions [21]. In a recent publication, the performance of dental students improved when they were exposed to the opinion of the AI before making their own decisions  [19]. However, the results demonstrated a significant gap between AI-assisted dental students’ performance and AI performance. This suggested that the protocol in which AI predictions were presented to participants might not be optimal. A possible cause for only moderate performance improvements observed in the group of dental students utilizing AI assistance might be related to a general lack of trust in AI.

This paper pioneers the idea of using AI to request second opinions for patients with advanced caries. For this study, we used an institutionally developed AI system to predict pulp exposure for patients diagnosed with advanced caries, following either stepwise excavation (SW) or non-selective excavation (NSE) treatment protocols [19]. An experiment was conducted to evaluate whether AI could introduce an improvement in the diagnosis and treatment planning process by identifying cases where dentists have potentially made a mistake and trying to correct such mistakes with the assistance of other dentists. The prediction of the AI system served as a trigger for the request for a second opinion from another clinician and was not exposed in the diagnosis process. It is hypothesized that this could benefit the decision-making process.

## Methods

### Database

The data used for training the AI model were diagnosed and treated teeth as previously published [22]. Included in the study were active extensive carious lesions. Using the international caries detection and assessment and international caries classification and management (ICDAS/ICCMSs™) systems [23], all cases were scored as ICDAS 5 and with a radiographical score (RS) of 5. Notably, the ICCMS™, as well as the ADA caries classification system (CCS) [24], separates the radiographical caries penetration depths in thirds. In this material, the carious lesions were further radiographically subdivided. Participants were enrolled if the primary extensive caries lesions were located in the pulpal quarter of the dentine. However, lesions that were extremely deep, penetrating through the entire thickness of the dentine, were excluded. Similarly, lesions that affected less than three-quarters of the dentine were also not included. The patients were all 18 years or older. In addition, they either had no pretreatment pain or mild to moderate pretreatment pain as provoked and confirmed by stimulation with cold or compressed air. Collectively, the patients were diagnosed with either healthy pulp or reversible pulpitis, and their periapical diagnosis was determined to be healthy [22]. Patients were excluded if they experienced continuous pain, showed no reaction to cold or electrical pulp tests, had attachment loss exceeding 5 mm, exhibited apical radiolucency, had any systemic condition preventing them from participating, were pregnant, or did not provide consent [22]. The study compared two types of treatments for deep carious lesions, namely SW and NSE. The SW procedure consisted of two sessions. During the first visit, selective removal up to soft dentine and non-selective removal of the peripheral demineralized dentine was performed until appropriate restoration. Following a period of 8 to 12 weeks, the patient returned for non-selective carious removal to hard dentine. The NSE procedure consisted of non-selective carious removal to hard dentine in one session. The cases were categorized as either successful or unsuccessful based on whether or not exposure occurred. Success cases presented unexposed pulps after excavation [22]. Consequently, the ground truth for our study was established based on the actual treatment outcomes. For each patient, the associated clinical information included gender, age, and self-reported pain levels (Table 1).

**Table 1:** The description of the patients and dental students who participated in this study

Characteristic	Value
**Clinical record**	
Patients (one tooth per patient)	290
Female	122
Male	166
Mild to moderate preoperative pain	103
No pain	187
Median age	29
Molars	161
Premolars	118
Incisors	8
Canines	3
**Randomized treatments** (ratio 1:1)	
Stepwise excavation group	142
Complete excavation group	148
**Treatment outcomes**	
Pulp exposure (Stepwise excavation)	24
Pulp exposure (Complete excavation)	42
**Participants**	
Number of participants	25
Female	22
Male	3
Fourth academic year	7
Fifth academic year	18

**Fig. 1:**
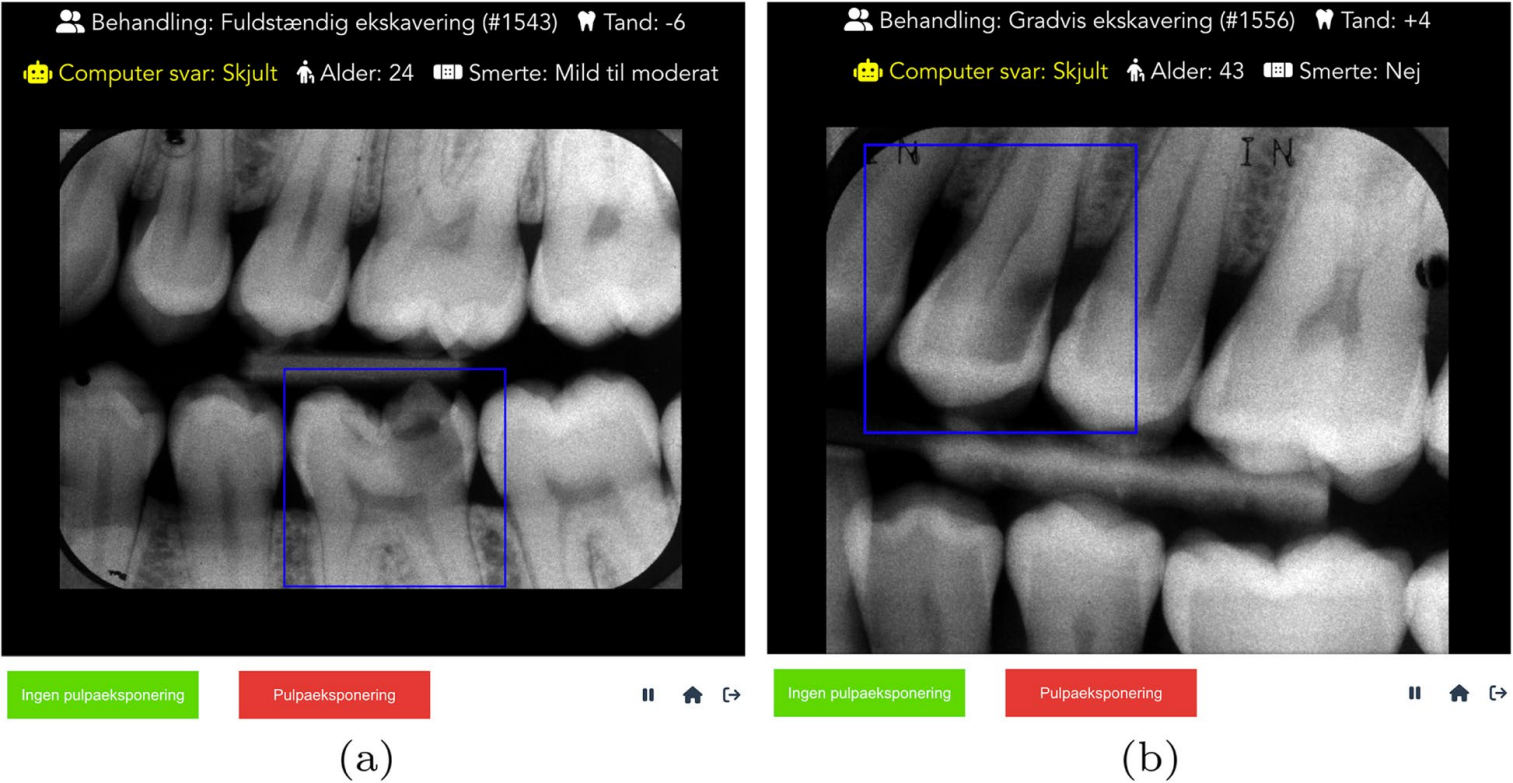
The experiment view of the platform designed to collect dentists’ predictions [19] Each sub-figure represents a different patient. All cases were extensive active stages (ICDAS 5, RC 5), with clinical details displayed at the top, including treatment type, tooth number, age, and pain information. The platform depicts all AI predictions as hidden during this experiment. A box highlights the affected tooth in each preoperative radiograph. The bottom bar includes two action buttons, representing “No pulp exposure” and “Pulp exposure” choices, respectively. It also features a progress bar indicating the participant’s current stage in the experiment. Patient (**a**) was treated with complete excavation and had pulp exposure. Patient (**b**) was treated with stepwise excavation and did not experience pulp exposure

### Experiment description

This project received ethical approval from the University of Copenhagen, Denmark (case: 504-0342/22-5000). The data collection was ethically approved by the joint Copenhagen and Frederiksberg ethics committees in Denmark (j.no: 03-004/03) [22].

**Fig. 2:**
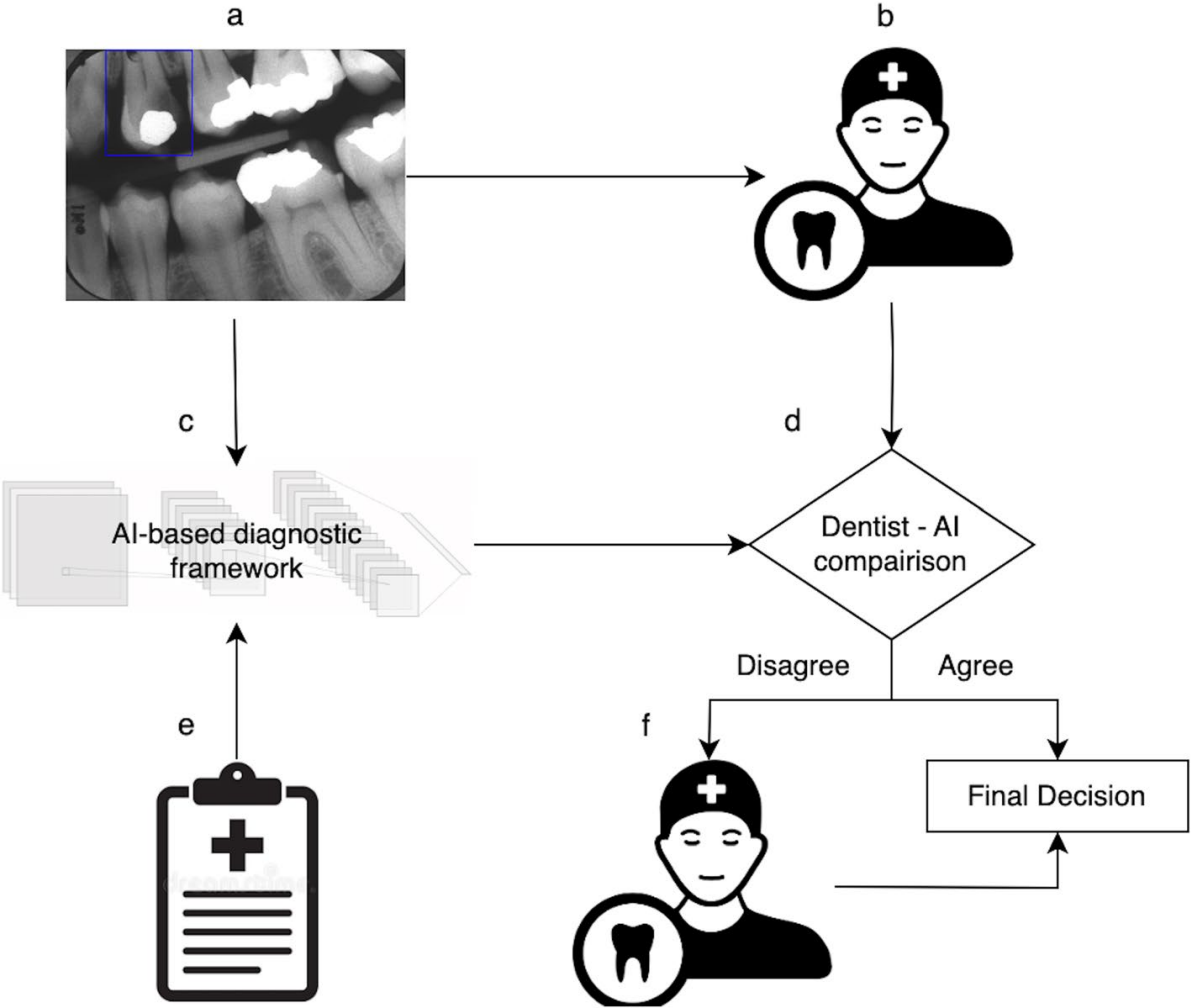
The AI-triggered second opinion framework. The input for this framework consists of a preoperative radiograph collected from a patient with advanced caries (**a**). Dentist 1 evaluates the case, determining whether the scheduled excavation treatment will result in pulp exposure or not (**b**). In the background of the framework, the AI system (**c**) also predicts the risk of pulp exposure using the preoperative radiograph (**a**) and clinical information (**e**). The prediction of the first dentist and AI are then compared by the framework (**d**). If the predictions do not agree, a second dentist (**f**) is consulted for their decision

The data and predictive framework were integrated into an AI-assisted dental experiment involving 25 dental students. The experiment was carried out using a custom-built diagnostic platform where the participants predicted the occurrence of pulp exposure following advanced caries treatments with and without AI assistance (Fig. 1). The web platform included two main views, namely the home page and the experiment page. The former presented the experiment, included a tutorial on how to navigate the experiment page, and noted key information regarding carious lesion treatments. The latter was responsible for recording the answers of the subjects. After starting the experiment, the web platform displayed dental cases one by one for the participants to classify. After analyzing a patient’s case, the participant was requested to click one of two buttons indicating whether they believed that the treatment would end in pulp exposure or not. As previously reported the performance of dental students did not improve significantly when seeing the opinion of the AI before making their decision [19]. In this paper, we explored a principally different idea, where the AI-generated decision was not displayed to the dental students. Instead, it functioned as a background service for soliciting second opinion, when the AI decision did not match the opinion of the dental students.

**Table 2:** The comparison of the dental student performance on pulp exposure prediction when they predict treatment outcomes alone (“Student” column), get AI-assisted second opinion support (“Second opinion” column), and get support from two other randomly selected dental students (“Majority voting” column). The results are reported in terms of the average F1-score (+- standard deviation when applicable). Both AI-assisted second opinion and majority voting results are calculated as the result of 1000 simulation trials

Participant	Student	Second opinion	Majority voting
1	0.476	** 0.608 ** ± ** 0.021**	0.579 ± 0.024
2	0.491	** 0.634 ** ± ** 0.024**	0.600 ± 0.025
3	0.519	** 0.576 ** ± ** 0.020**	0.545 ± 0.024
4	0.546	** 0.590 ** ± ** 0.020**	0.560 ± 0.025
5	0.549	** 0.672 ** ± ** 0.019**	0.615 ± 0.024
6	0.562	** 0.605 ** ± ** 0.027**	0.565 ± 0.026
7	0.565	** 0.611 ** ± ** 0.022**	0.569 ± 0.024
8	0.572	** 0.663 ** ± ** 0.020**	0.611 ± 0.025
9	0.576	** 0.646 ** ± ** 0.022**	0.615 ± 0.025
10	0.580	** 0.623 ** ± ** 0.022**	0.612 ± 0.025
11	0.581	** 0.626 ** ± ** 0.019**	0.607 ± 0.022
12	0.584	** 0.649 ** ± ** 0.022**	0.615 ± 0.023
13	0.592	** 0.632 ** ± ** 0.021**	0.607 ± 0.022
14	0.593	** 0.669 ** ± ** 0.024**	0.630 ± 0.024
15	0.597	** 0.659 ** ± ** 0.023**	0.612 ± 0.025
16	0.603	** 0.642 ** ± ** 0.028**	0.615 ± 0.028
17	0.603	** 0.649 ** ± ** 0.020**	0.620 ± 0.024
18	0.613	** 0.644 ** ± ** 0.020**	0.598 ± 0.023
19	0.615	** 0.683 ** ± ** 0.021**	0.620 ± 0.023
20	0.615	** 0.640 ** ± ** 0.022**	0.596 ± 0.023
21	0.632	** 0.658 ** ± ** 0.023**	0.603 ± 0.024
22	0.635	** 0.702 ** ± ** 0.018**	0.650 ± 0.023
23	0.637	** 0.665 ** ± ** 0.026**	0.648 ± 0.026
24	0.653	** 0.693 ** ± ** 0.019**	0.643 ± 0.026
25	0.670	** 0.686 ** ± ** 0.020**	0.659 ± 0.026

### Experiment design

After collecting predictions from all participants, we simulated a second opinion experiment. To exemplify, consider the classification of case *J* performed by student *A* from the set of students *D*. The prediction of participant *A*, denoted as $q_{J}^{A}$, was compared with the AI’s classification, denoted as $q_{J}^{AI}$. If $q_{J}^{A}$ aligned with $q_{J}^{AI}$, it was recorded as the final response for that case. In instances where $q_{J}^{A}$ differed from $q_{J}^{AI}$, a dentist B was randomly selected from the set $D \setminus \{A\}$, and their response, $q_{J}^{B}$, was recorded as the final response for case *J*. It is important to note that no participant had access to the AI classifications; they were only used to trigger second opinions. Figure 2 depicts the AI-triggered second opinion framework.

### Model

We developed a multi-path neural network including a convolutional module and a fully connected module to classify the deep caries treatment outcome as success or failure depending on the status of the pulp. The input of the convolutional module consisted of preoperative bitewings featuring the carious lesions. The input of the fully connected module included clinical features such as the distance from the lesion to the pulp, the patient’s age, and the patient’s pain status before the intervention. To extract information from image input we used a 50-layer Residual Network. It is a feed-forward convolutional neural network that consists of stacked residual blocks. The architecture relies on skip-connections to facilitate the training process and learn very deep representation [25]. We leveraged transfer learning by fine-tuning parameters tailored to the ImageNet database. The output of the Residual Network was an $f_i$-dimensional image embedding which was concatenated with $f_c$-numerical features extracted from the target case. The resulting ($f_i$+$f_c$)-dimensional case representation was followed by a linear layer with binary classification output. We utilized standard neural network components to simplify the repetition of the second opinion experiment. The code including the algorithm can be found at the following GitHub repository: https://github.com/tudordascalu/pulp-exposure-classification.

The radiographs were subjected to a set of transformations during the framework training. The teeth of interest were cropped using manually drawn bounding boxes because there were patients with multiple teeth affected by carious lesions. Then, the cropped images were resized using zero padding to the size of the largest box (958 x 872) and subsequently down-scaled by a factor of 2. The training set was augmented through affine transformations, brightness, and contrast adjustments.

The numerical features consisted of clinical data and morphometric information extracted from the preoperative radiographs. The morphometric information described the connection between the lesion and the pulp, a critical aspect influencing the treatment outcome. We computed the Euclidean distance between the closest pair of points between the expert annotations of pulp and carious lesions semi-automatically, from caries and pulp annotations. The final output consisted of a continuous value ranging between 0-1, denoting the probability that the advanced caries treatment for a given tooth ended up in exposure to the pulp.

### Statistical analysis

The main metric reported in the present work corresponds to the macro-average F1-score, equivalent to the harmonic mean of precision and recall, treating both classes equally important. The classification outcomes are categorized as follows: true positives (TP), true negatives (TN), false positives (FP), or false negatives (FN). True positives correspond to instances where the model correctly predicts pulp exposure. True negatives correspond to instances where the model correctly predicts the absence of pulp exposure. False positives are instances where the model erroneously predicts pulp exposure when there is none. False negatives are instances where the model fails to predict pulp exposure when it is present. Precision is calculated as the ratio of true positive predictions to all positive predictions: $\frac{TP}{TP + FP}$. Recall, on the other hand, is the ratio of true positive predictions to all actual positive cases: $\frac{TP}{TP + FN}$. For assessing statistical significance, we employed Student’s T-test (with the Bonferroni correction for multi-group comparisons), and calculated confidence intervals (CI). Tests with p-values less than 0.05 were considered statistically significant. Additionally, we used the Pearson correlation coefficient to examine the relationship between variables. We conducted simulations using Python’s integrated “random” module, executing a total of 1000 trials for each experiment.

## Results

The present study consisted of multiple sessions in the span of two weeks. It included $n=25$ participants, out of which 22 were female and 3 were male (Table 1). All were dentistry students in their 4th and 5th years of education at the Department of Odontology at the University of Copenhagen, Denmark. The students passed the course needed to understand the concept of advanced caries pathology and treatment (Cariology, Advanced Direct Restoration 2, and Advanced Endodontics 2).

The AI framework defined in the previous section was trained and tested on a machine with a Titan X GPU with 12GB of memory. The model parameters were optimized for 50 epochs using the RMSprop algorithm, with a learning rate of $10^{-4}$ and an L2 regularization parameter set to $10^{-8}$. The learning rate was reduced after stagnating for 10 epochs by a factor of $10^{-1}$. The data set was split into mini-batches of size 8. It was subjected to a set of transformations. The images were randomly flipped in the horizontal direction, rotated with an angle ranging from -20 to 20 degrees, translated in the X, and Y directions by a factor of 0.1, and scaled by a factor ranging between 0.8 to 1.2. Gaussian noise with a mean of 0 and a standard deviation of 0.05 was added. Perspective changes were applied using a degree of distortion set to 0.1.

The database included 290 cases in patients aged between 18 and 89. Of the active extensive carious lesions, ICDAS 5 included in the study, 96.2% were approximal lesions. In total, 142 patients were randomly treated using SW, and the remaining 148 patients were treated using NSE. The patient collection included 166 males and 122 females. Mild to moderate pain levels prior to the treatment were reported in 103 cases (35%). In the SW arm, there were 25 pulp exposures (19%), compared to 43 pulp exposures (29%) in the NSE arm. Bitewing radiographs were recorded for each patient. The radiographs were acquired by various scanners with resolutions ranging from 453x374 to 1561x1945. The images represented digitalized analog X-rays. A dental professional created bounding boxes around the teeth of interest, with sizes ranging from 162x155 to 958x873.

The average decision time per patient was 12.23 ± 6.03 sec (± standard deviation) for cases without clinical information (type 1) and 12.36 ± 6.11 sec (± standard deviation) for cases with clinical information (type 2) cases. The average F1-score of dental students was 0.586. The AI system achieved an F1-score of 0.71. In total, the participants agreed with AI in 2539 readings (agreement subset) and disagreed in 1085 cases (disagreement subset). In the disagreement subset, the patients had an average age of 32 years. Among these patients, 43% experienced preoperative pain. Additionally, 54% of them underwent SW, while 46% underwent NSE. Each sample from the disagreement subset was submitted for a second opinion in a 1000-trial simulation. On average, the dental students’ F1-score for the disagreement subset was 0.289 (95% CI: 0.257-0.321). By implementing the second opinion framework, their performance improved significantly, with an average F1-score for the disagreement subset of 0.468 (95% CI: 0.447-0.49; $P < 0.05$).

The participants achieved F1-scores of 0.725 for cases in agreement with the AI and 0.295 for cases in disagreement. Assuming that there might be demographic or clinical differences influencing the difficulty of the cases, we compared demographic and clinical factors for cases where participants and AI were more likely to agree and disagree. We divided the cases into high agreement (HA) if more than 70% of the participants had the same diagnosis as the AI solution, and low agreement (LA), otherwise. This resulted in 171 cases for the HA group, and 119 cases for the LA group. We found no significant difference between the two groups in terms of gender ($P>0.05$). The proportions of females were 44.4% and 39.5% in HA and LA, respectively. Similarly, no significant correlation between agreeableness levels and treatment type was found ($P>0.05$). The proportions of patients treated with SE were 42.9% and 53.2% in HA and LA, respectively. There was a significant correlation between agreeableness levels and preoperative pain levels ($P<0.05$). The proportions of patients without preoperative pain were 69.6% and 57.1% in HA and LA, respectively. Furthermore, there was a significant difference in the accuracy of the AI on the groups HA and LA ($P<0.05$). The accuracy of the AI model on the HA group was 0.84, while the accuracy of the model on the LA group was 0.697. The patients in the HA group were significantly older than the patients in the LA group ($P<0.05$). The average ages of the patients were 34.6 (95% CI: 32.796-36.42) and 31.16 (95% CI: 29.542-32.777) in HA and LA, respectively.

To grasp the practical benefits of our second opinion framework, we carried out 1000 Monte Carlo simulations for each of the 25 dental students involved in our study. For every simulation, we processed all responses from a participant, replacing them with hybrid predictions generated by the second opinion framework. We observed a significant increase in the average F1-score of the dental students from 0.586 without second opinions to 0.645 when second opinions were requested. The Pearson correlation coefficient between the F1-score improvement and dentist performance was $-0.734$, indicating that the second opinion framework provided more benefit for participants with lower baseline performance. Nevertheless, even the students with the best initial performance significantly improved their performance with the help of a second opinion. The performance of the initial dental student improved in 96.3% out of the total of 25000 second opinion random trials. The dental students with the second opinion framework outperformed two experienced dentists on the same data set, who achieved F1-scores of 0.595 and 0.598, respectively [19].

We implemented an alternative prediction pipeline where the initial decision from a dental student was always supplemented by two additional evaluations from randomly selected dental students. The final decision was determined by the most prevalent opinion among the three, i.e. the majority vote protocol. Table 2 displays aggregated performance metrics for each dental student, alongside the simulation results of the second opinion and majority vote protocols. The average F1-score reached by the participants was 0.586 (95% CI: 0.567-0.605). The performance increased for the second opinion framework to the average F1-score of 0.645 (95% CI: 0.632-0.658). The average F1-score for the majority vote approach was 0.608 (95% CI: 0.596-0.62). To test the significance of the results, we employed pairwise paired t-tests with Bonferroni correction. The results indicated significant differences between the second opinion method and both the dental students’ performance and the majority vote method ($P < 0.05$).

**Fig. 3:**
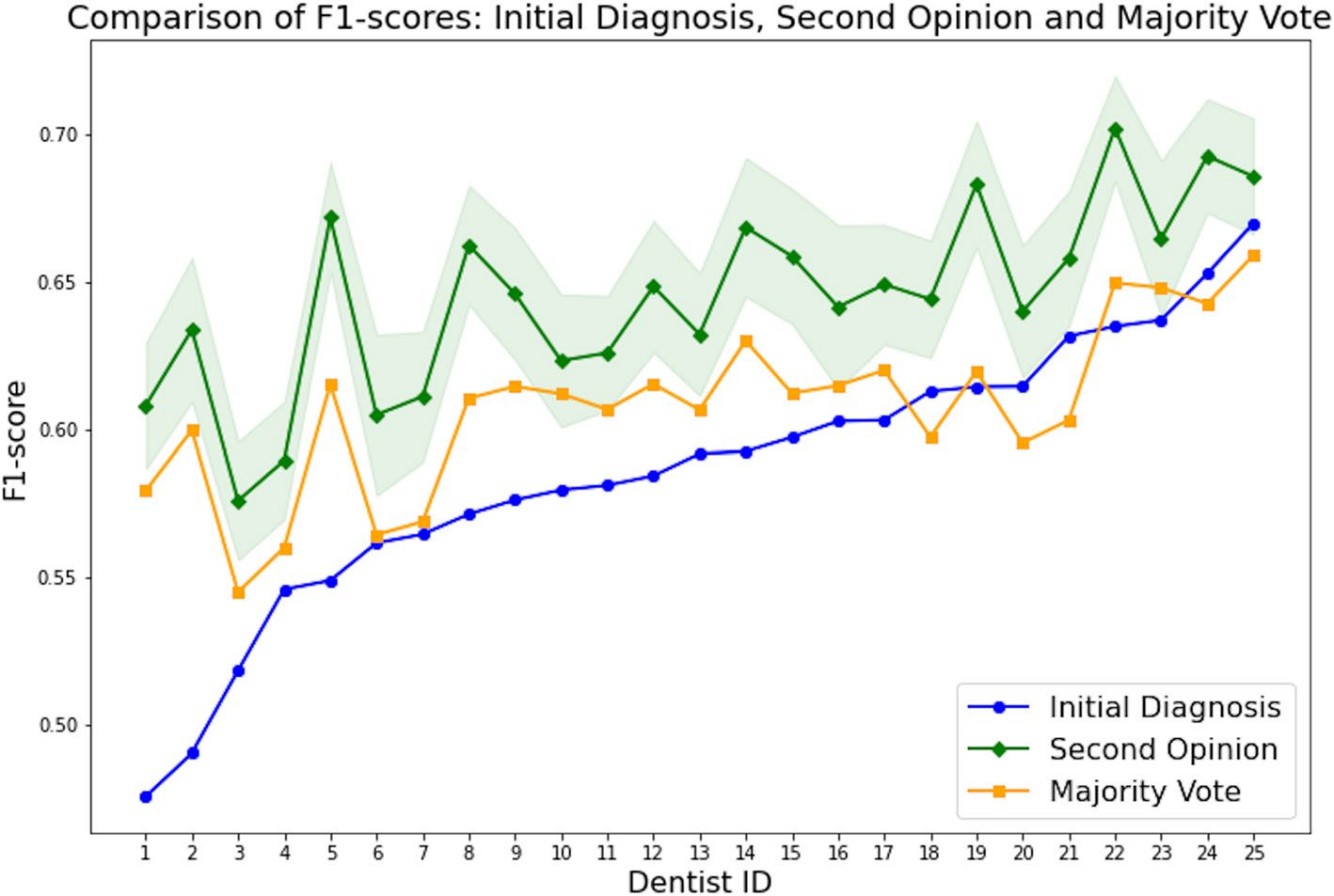
The carious treatment outcome prediction summary for all dental students with and without AI-assisted second opinion in terms of F1-score. Each tick on the X-axis represents an individual dental student, for whom we computed their individual performance without any assistance (blue) and their performance with the AI-triggered second opinion (green). The shadowed area around the AI-triggered second opinion results corresponds to the +-one standard deviation of F1-score computed from 1000-second option simulation trials. For comparison, we ran a majority voting experiment, when the first dental student was assisted by two random dental students so that each decision was the product of a three-dentist-majority vote. The orange curve is the average majority voting performance computed for each dental student using 1000 simulations

## Discussion

Trust in intelligent systems among clinical specialists remains a core challenge in the successful integration of AI in healthcare settings [18]. Prior research has shown that domain experts’ confidence in intelligent systems drops after observing a few critical errors made by such systems [26, 27]. We also documented trust-related performance issues in our previous dental experiments [19]. Despite being informed about the algorithm’s accuracy, the experienced dentists participating in the dry runs of our experiment maintained the same performance levels regardless of the AI assistance. Additionally, a certain level of distrust was observed among the dental students participating in the experiment, even after being explicitly informed that the AI’s performance was similar to that of dental experts. In this study, we addressed this trust issue by exploring a principally different approach, wherein AI did not directly influence dentist decision-making. Instead, it served as a background tool within the diagnostic pipeline, requesting second opinions when the model detected the need. In our second opinion protocol, the initial decision made by a dentist was accepted if it aligned with the AI prediction. If not, a second dentist was consulted to provide a diagnosis, which was considered the final decision. The distinction between our second opinion protocol and a conventional first-second opinion diagnostic pipeline lies in the fact that the AI model requests a second opinion. The clinical significance of the task undertaken in the present study corresponds to the fact that we aim to minimize treatment variation, particularly when it comes to deciding whether to expose the pulp or not. There is substantial evidence indicating that pulp exposure can be avoided in well-defined deep lesions with a radiographical penetration into the pulpal quarter. Our system was evaluated in the context of the predictive assessment of pulp exposure risk in treating advanced carious lesions. The approach to managing advanced caries varies significantly among clinicians, especially concerning the decision to expose the pulp during treatment. Evidence strongly suggests that it is possible to avoid pulp exposure in cases of advanced lesions with well-defined penetration into the pulpal quarter [10, 22]. Conversely, in some regions, the threshold for considering pulp exposure begins at a penetration depth of one-third of the dentine [11], highlighting a discrepancy in clinical practice worldwide. Therefore, the clinical implications of our AI-based tool have been to improve the selection of the best treatment option for advanced caries excavation and the pulp close information from radiographs to an extent not used before.

The results of the simulation indicated that the second opinion predictions altered the initial decisions in 43.5% of the cases. When a dentist treats a patient with advanced caries that poses a risk of pulp exposure, the following courses of action are generally considered: if the initial treatment was NSE and the AI predicted pulp exposure, the patient might be recommended to undergo a less invasive treatment like selective carious tissue removal or if not feasible SW [16] as tested here; if the initial treatment was SW and the AI predicted pulp exposure, the patient might be advised to undergo a more aggressive pulp treatment. Using these considerations, 24.9% of cases initially predicted to result in pulp exposure and directed for less invasive or a more aggressive treatment by the first dental student were correctly switched to a less invasive or more aggressive treatment, respectively, while 4.9% were incorrectly switched.

In addition to the quantitative analysis of challenging cases comparing the high and low agreement groups, we performed a qualitative analysis by reviewing the cases with poor participant performance and identified potential clinical and image features that made these cases challenging to analyze. Figure 1 displays two such cases, with misdiagnosis rates of 100% (a) and 76% (b). The patients (due to the randomization within the original clinical trial [22]) underwent NSE (a) and SW (b), with different outcomes: pulp exposure was observed in patient (a), while patient (b) had no pulp exposure. For patient (a), a potential factor influencing the prediction difficulty may be the relatively small radio-opaque line that separates the lesion from the pulp chamber, which complicates the lesion depth assessment. For patient (b), the angle of the radiograph might have influenced the participants’ predictions, leading them to anticipate pulp exposure despite a clearer separation between the lesion and the pulp.

The majority voting strategy was evaluated following the same simulation framework as second opinion. The mean F1-score exhibited a minor improvement from the baseline value of 0.586 to 0.608. There were two main concerns associated with majority vote. First, it was ineffective as it required three dentists for each diagnosis. Second, it led to a convergence of prediction performance toward the group’s average. In other words, the majority vote benefited the participants with lower performance by improving their prediction accuracy towards the mean performance of the dental students. On the other hand, it negatively affected dental students with significantly above-average performance. Figure 3 shows that 5 dental students in the group experienced a decline in performance when employing a majority vote.

Our AI model attained an F1-score of 0.71 in predicting pulp exposure following the excavation of advanced caries. The model’s performance was partially limited due to its inputs. Bitewing radiographs, which offer 2D visualizations of the affected teeth, can be compromised by noise and may present occluded or distorted representations (either foreshortened or elongated) of the three-dimensional structures of interest [28]. Although not flawless, the AI model demonstrates promising utility, especially considering its superior performance compared to dental clinicians [19]. The second opinion framework mitigates the risks associated with deploying such a system in a clinical environment. Fully automated decision systems demand outstanding performance for adoption in clinical settings, as even a small percentage of algorithmic failures could negatively affect the patients’ heath. These high-performance requirements hamper the clinical adoption of AI-automated decisions. In our diagnostic protocol, AI-generated predictions were not disclosed to the dentists, which could not affect their decisions. Such a framework could have potentially moderate requirements on the AI performance to be applicable in clinical practice. We conducted a series of experiments by artificially adjusting the AI’s accuracy. This provided us with the lower and upper-performance bounds of the second opinion framework when applied to the task of pulp exposure prediction, based on the accuracy of the AI. For an AI model with a balanced accuracy of 0.6, which falls below the balanced accuracy of dental students (0.621), all non-outlier participants’ performances exceeded the mean F1-score of a dentist, which is 0.586. Using a perfect AI classifier, the mean F1-score across 1000 iterations was 0.729 (95% CI: 0.717-0.742). The participants’ prediction capabilities constrained the maximum performance attainable by the framework. While the second opinion framework may ease AI performance requirements for integration into clinics, a limitation is that perfect AI performance doesn’t ensure flawless treatment planning. Instead, it ensures that a second opinion is solicited in instances where dentists make mistakes. In essence, the second opinion framework represents a risk-averse approach to integrating intelligent systems into dental practice.

The second opinion framework could be integrated into existing dentistry software for the management and visualization of clinical records. The dentist attending a patient could input the treatment plan into our module. This plan would be compared with the AI’s recommendation in the background. If there’s a discrepancy between the dentist’s plan and the AI’s suggestion, the patient’s data would be shared with a network of affiliated dentists. The treatment plan proposed by the first responding dentist from this network would then be provided as an enhanced recommendation to the initial dentist. Furthermore, this tool may also be integrated into dental schools, promoting collaboration and critical discussion among dental students, particularly in complex cases. The University of Florida’s research in integrating AI into dental education provides valuable insights for such implementations [29]. Their study examines the adoption of AI from multiple perspectives, including structural, human resource, political, and symbolic dimensions. It underscores AI’s potential not only in attracting more students and enhancing the curriculum but also in reducing the workload of faculty and staff. This holistic approach to AI integration in dental schools can serve as a model for how our second opinion framework might be utilized in an educational setting, promoting a more collaborative and technologically advanced learning environment.

A potential application of our platform could focus on reducing treatment variability. Reducing treatment variability in predoctoral clinical programs can be achieved by ensuring accurate procedure-diagnosis pairing [30]. The implementation of electronic health records facilitates this process [30]. However, the treatment of advanced lesions presents a dilemma, as the same diagnoses have historically led to a range of treatment invasiveness [31], a trend that continues today [12]. Even though systematic reviews and reports recommend a less-invasive approach [10, 16, 32], the process of transferring research into practice is slow [33]. Taking controlled research data and recent guidelines into account, it seems very likely that pulp exposure could be avoided when using a less invasive carious tissue removal procedure. In our platform, students encountered extensive carious lesions that were uniformly diagnosed with a well-defined penetration depth in the pulpal quarter. They were asked to predict the outcome based either on an invasive approach versus a less invasive approach. In the future, a similar platform could enhance training in a procedure-diagnosis pairing methodology, as explored by White et al. [30]. The task integrated into the platform could be reformulated to classify extensive caries within the region of the pulpal quarter into the deep lesion (DL) 1 located in the pulpal 2/3, DL 2 within the pulpal quarter, and DL 3 extending throughout the entire quarter [34]. It is also important to note that the presented AI-initiated second opinion framework is not limited to specific caries treatment technologies or treatment outcomes and rather represents a universal approach for integrating AI for dental decision-making assistance.

## Conclusion

This paper introduces a framework for incorporating AI solutions into clinical dental practice without influencing dentists’ decisions with AI predictions. We designed an AI-triggered second opinion diagnosis method. The outcomes of the experiments revealed an increase in the average F1-score from 0.586 to 0.645, confirming the hypothesis that the second opinion framework might benefit the decision-making process.

## Data Availability

The datasets generated during and/or analyzed during the current study are not publicly available due to privacy concerns but are available from the corresponding author on reasonable request.
